# Detection of rare disease variants in extended pedigrees using RVS

**DOI:** 10.1093/bioinformatics/bty976

**Published:** 2018-11-30

**Authors:** Thomas Sherman, Jack Fu, Robert B Scharpf, Alexandre Bureau, Ingo Ruczinski

**Affiliations:** 1 Department of Biostatistics, Johns Hopkins Bloomberg School of Public Health; 2 Department of Oncology, Johns Hopkins School of Medicine, Baltimore, MD, USA; 3 Département de Médecine Sociale et Préventive, Université Laval, Québec, QC, Canada; 4 Centre de Recherche CERVO, Québec, QC, Canada

## Abstract

**Summary:**

Family-based sequencing studies enable researchers to identify highly penetrant genetic variants too rare to be tested in conventional case-control studies, by studying co-segregation of variant and disease phenotypes. When multiple affected subjects in a family are sequenced, the probability that a variant or a set of variants is shared identical-by-descent by some or all affected relatives provides evidence against the null hypothesis of complete absence of linkage and association. The Rare Variant Sharing software package RVS implements a suite of tools to assess association and linkage between rare genetic variants and a dichotomous disease indicator in family pedigrees.

**Availability and Implementation:**

RVS is available as open source software from the Bioconductor webpage at https://bioconductor.org/packages/release/bioc/html/RVS.html.

**Supplementary information:**

Supplementary data are available at *Bioinformatics* online.

## 1 Introduction

Sequencing distant relatives is an established approach to identify causal variants in Mendelian disorders, but is also increasingly applied to identify genetic risk variants in complex disorders. When familial aggregation of a phenotype is observed at a rate much higher than the prevalence in the general population, one possible explanation can be the segregation of a rare and highly penetrant disease variant. We recently devised a statistical framework for such a setting, based on the notion that sequencing DNA in extended multiplex families can help to identify such high penetrance disease variants too rare in the population to be detected through tests of association in population-based studies (Bureau *et al.*, 2014a). Specifically, when several affected subjects per family are sequenced, evidence that a rare variant may be causal can be quantified from the probability of sharing alleles by all affected relatives given it was seen in any one family member under the null hypothesis of complete absence of linkage and association. We presented a general framework for calculating such sharing probabilities when two or more affected subjects per family are sequenced, and show how information from multiple families can be combined by calculating a *P*-value as the sum of the probabilities of sharing events at least as extreme (Bureau *et al.*, 2014a). Sequencing three affected second cousins from a family with multiple oral cleft cases, we successfully employed this approach to identify a causal nonsense mutation in the gene CDH1 (Bureau *et al.*, 2014b). Recently, we extended the methodology to allow for gene- or region-based tests, and partial sharing of variants (Bureau *et al.*, 2018). We also investigated sharing of rare copy number variations, and implemented a global test for an enrichment of variant sharing (Fu *et al.*, 2017). The novel Rare Variant Sharing software package RVS is an open source implementation of these methods. The software builds on existing infrastructure for family-based genetic studies and probability propagation in graphical networks (probabilistic expert systems) to calculate sharing probabilities in pedigrees. RVS differs from other methods commonly employed for sequence data analyses in pedigrees as no parental or founder data is required (which can be of great advantage, as it can be difficult to obtain DNA from past generations), only affected subjects are sequenced, and estimates of population variant frequencies are not required (Supplementary Material).

## 2 Features

### 2.1 Calculating sharing probabilities and *P*-values

**Fig. 1.**
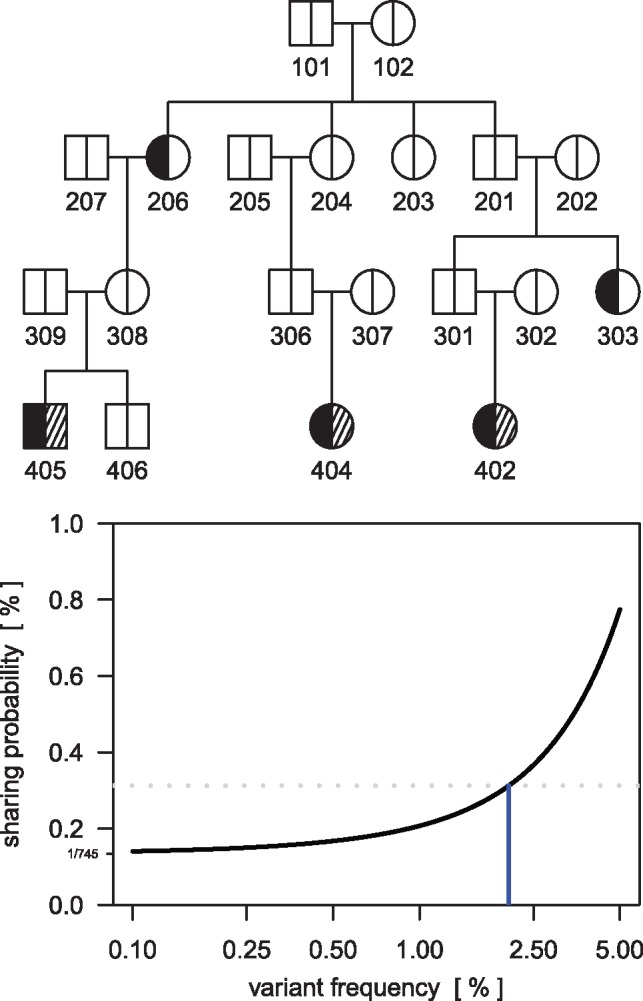
**Top:** Pedigree with three affected second cousins sharing a rare nonsense variant in gene CDH1. Affected subjects in this family are indicated by filled left parts of the symbols. Individuals 402, 404 and 405 were sequenced, indicated by shaded right parts of the symbols. **Bottom:** The exact sharing probabilities (in percent, y-axis) as a function of variant frequency (in percent, x-axis) for the three second cousins. The RVS sharing probability calculated under the assumption of ‘no IBS without IBD’ is 1/745. The dotted gray line indicates significance after a Bonferroni correction for 16 potentially causal variants analyzed (Bureau *et al.*, 2014b). A population allele frequency of up to 2.05% (vertical line) would have still yield significant variant sharing

The primary function RVsharing of the RVS package is to calculate exact variant sharing probabilities in extended multiplex pedigrees. The genetic variant can be a single nucleotide variant (e.g. Bureau *et al.*, 2014b) or a copy number variant (e.g. Fu *et al.*, 2017). Given the variant has been observed in the family, what is the probability that it is shared identical-by-descent by all sequenced affected relatives? For a single family, this probability can be interpreted directly as a *P*-value for testing the null hypothesis of absence of linkage and association. For example, the sharing probability of the CDH1 nonsense mutation among the three second cousins with oral clefts is 1/745 (Fig. 1 top, and Bureau *et al.*, 2014b). For variants seen in more than one family, the *P*-value can be obtained as the sum of the probability of events at least as extreme as the observed sharing, and can be calculated using the function multipleFamilyPValue. The function multipleVariantPValue generalizes multipleFamilyPValue across multiple variants. This function also provides a filtering option based on the ‘potential *P*-values’, which is the significance a variant could achieve if it was shared by all affected, and usually only variants that could pass a multiple comparisons correction are retained in an analysis (Bureau *et al.*, 2014a). Of note, even if no individual variant might achieve a significant *P*-value, it is possible that all variants considered jointly exhibit more sharing than can be explained by random chance (Fu *et al.*, 2017). The enrichmentPValue function can compute a single *P-*value to test for such an enrichment of variant sharing. For the analysis of a genomic region such as a gene, the function RVgene implements the *P*-value computation described in Bureau *et al.* (2018), and also offers a partial sharing test which considers probabilities of sharing among a given subset of the affected subjects, calculated using the carriers argument in the RVsharing function. Both multipleFamilyPValue and RVgene take as input objects of class snpMatrix storing single nucleotide variant genotypes extracted from ped or Variant Call Format (vcf) files by functions from the VariantAnnotation and snpStats Bioconductor packages.

### 2.2 Follow-up and sensitivity analyses

The two key assumptions in the variant sharing methodology are that the damaging variant is sufficiently rare in the population that only one founder introduces it into the pedigree (i.e. identical-by-state implies identical-by-descent) and that the founders are unrelated. Violations of either of these assumptions inflate the actual sharing probability, and thus can lead to false positive identifications. The first assumption is critical when the allele frequency of the damaging variant is unknown, which is commonly the case. Using the argument alleleFreq in the RVsharing function allows for a fast and convenient sensitivity analysis of the rare variant assumption. Exact sharing probabilities in the pedigree can be calculated for any assumed population rare variant allele frequency by making extensive use of the underlying probabilistic expert system, propagation the genotype probabilities from the founders to the sequenced subjects. For the CDH1 nonsense mutation (Bureau *et al.*, 2014b) a population allele frequency of up to 2.05% would still have yielded a significant sharing probability under a Bonferroni correction, where a total of 16 potentially causal variants were assessed (Fig. 1, bottom). When founders of the pedigree are related, computing the variant sharing probabilities becomes even trickier. The correction described in Bureau *et al.* (2014a) uses the mean kinship coefficient among founders passed as argument to the RVsharing function using the kinshipCoeff parameter. When estimates of kinship coefficients are only available for descendants, the ComputeKinshipPropCoef function can be used to infer the mean kinship coefficient of the founders. Any complex relationships among founders can be specified using the argument founderDist. In the RVS implementation, sharing probabilities in pedigrees with inbreeding can be calculated directly and no longer require the gene-dropping approach described in Bureau *et al.* (2014a). Gene dropping remains available by specifying the nSim argument in RVsharing.

## 3 Conclusion

Rare genetic variant sharing analysis is a powerful approach to identify causal variants underlying complex disease risk. The open source RVS Bioconductor package provides convenient functionality to carry out such analyses with excellent scalability (Table 3 in Bureau *et al.*, 2018, and Supplementary Material). To facilitate reproducible research, a software vignette with an example of a workflow is provided at the Bioconductor website.

## Supplementary Material

bty976_Supplementary_Data
