# Supplementary material for: Detection of rare disease variants in extended pedigrees using RVS
Source: Bioinformatics. 2018 Nov 30;35(14):2509–11. doi: 10.1093/bioinformatics/bty976 (PMC6612888; doi:10.1093/bioinformatics/bty976)
Supplement: bty976_Supplementary_Data [file bty976_supplementary_data.pdf]

September 19, 2018

Computational Considerations

A Benchmark

Calculating sharing probabilities among affected subjects is very efficient even in very large pedigrees. To create a benchmark, we generated pedigrees with a single founding pair, three subsequent generations with two children per couple, and n subsequent generations with a single child per couple. We assume the final generation of 8 children is sequenced (Supplementary Figure 1, left; $n = 2$ for a total of 54 subjects). The benchmark for the standard *RVsharing* calculation on a laptop with a Intel i7-8650U @ 1.90GHz processor indicates that calculations are virtually instantaneous for any reasonably sized pedigree of this type (Supplementary Figure 1, right), and takes about 60 seconds to run on a pedigree with 614 subjects, 304 founders, and 8 sequenced subjects ($n = 37$). In the section below we argue that the run time scales with the square of the number of subjects.

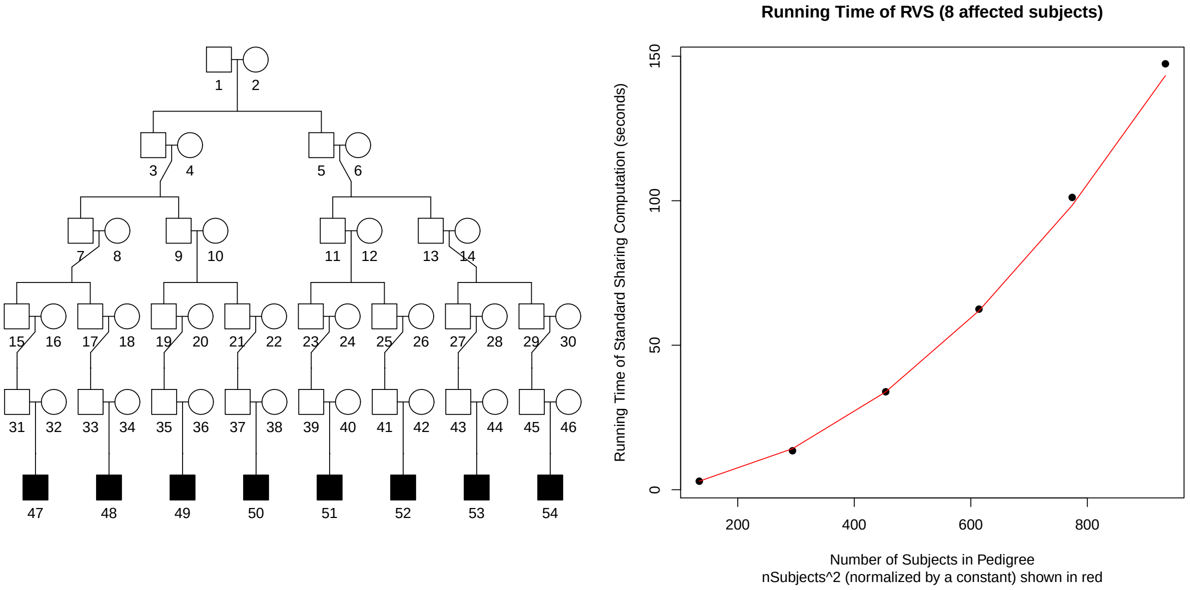

Supplementary Figure 1: [LEFT] example of a multi-generation pedigree without inbreeding, used in the benchmark study. [RIGHT] run time of the *RVsharing* calculation on a standard laptop (y-axis) as a function of pedigree size (x-axis).

Computational Complexity

The computational complexity of *RVsharing* depends on the structure of the underlying Bayesian network representing the pedigree, which dictates how expensive each marginal probability calcu-

lation will be. For general Bayesian networks with N total nodes, we can compute the marginal probability of a single node in $O(N \cdot 3^{|C|})$, where $|C|$ is the size of the largest clique (complete subgraph) in the underlying network. In general, $|C|$ can scale linearly with the number of nodes, however, pedigrees provide limitations on the amount of inter-connectedness that can be present in the network. For instance, a single node can only have two parent nodes and the children of a single node cannot (usually) be connected. In practice this is enough to effectively limit $|C|$ to a constant (it is possible to draw pedigree structures that grow $|C|$ linearly with N, however they rely on inbreeding in patterns not seen in the real world). Therefore, each marginal probability computation takes $O(N)$ time where N is the number of subjects in the pedigree. We present a more detailed argument why $|C|$ can be treated as a constant in the next section.

The number of marginal probability computations required depends on the parameters passed to *RVsharing*. If a minor allele frequency is given, there are N_s computations, where N_s is the number of sequenced subjects. In the standard variant sharing analysis where we assume only one founder introduces the variant, we need to condition on each of the founders in turn, resulting in $N_f \cdot N_s$ computations (when we correct for relatedness among founders, we have to condition on each pair of founders in the approximation, resulting in $N_f^2 \cdot N_s$ computations). Thus, the standard *RVsharing* calculation runs in $O(N \cdot N_f \cdot N_s)$, which in practice can be approximated as $O(N^2)$, since $N_f \leq N/2$, and typically $N_s \ll N$.

Size of the Largest Clique

In pedigrees without inbreeding (typically, the vast majority of pedigrees in our sequencing studies) we can make the precise statement that $|C| = 3$. The first two steps in the belief propagation algorithm are moralization and triangularization of the underlying network. The Bayesian network representation of a pedigree is a directed acyclic graph, where parent nodes have a directed edge towards their children. The moralization process makes all edges undirected and adds an edge between parents. Making the edges undirected or adding an edge between parents can create a cycle larger than three is if two parents are related. Since we are assuming no inbreeding and no marriage loops, we can safely say that no cycles are created besides the parent-parent-child cycles that are created for each child node. The final step is triangularization, however, since we have no cycles larger than three, we already have a triangular graph. Therefore the largest clique (complete subgraph) has size three.

When inbreeding or marriage loops are present in the pedigree we unfortunately can not make a general statement about $|C|$. Based on observations that real world pedigrees have $|C| < 7$, we explain the pattern counterexamples have, and show that it is an unrealistic scenario (i.e. represent pedigrees we would not draw samples from for sequencing). One counterexample is a pedigree with an entire generation inter-marrying multiple times (Supplementary Figure 2). If there are $2N$ cousins (N males and N females) where each of the N males mates with k females (and vice versa), then $|C|$ will grow linearly with k. The key is that the running time is exponential in the number of same-generation partners each subject has - not the number of subjects who have a same-generation partner. We believe all pedigrees that require exponential running time follow this pattern, and as such are highly contrived and do not represent real pedigree structures.

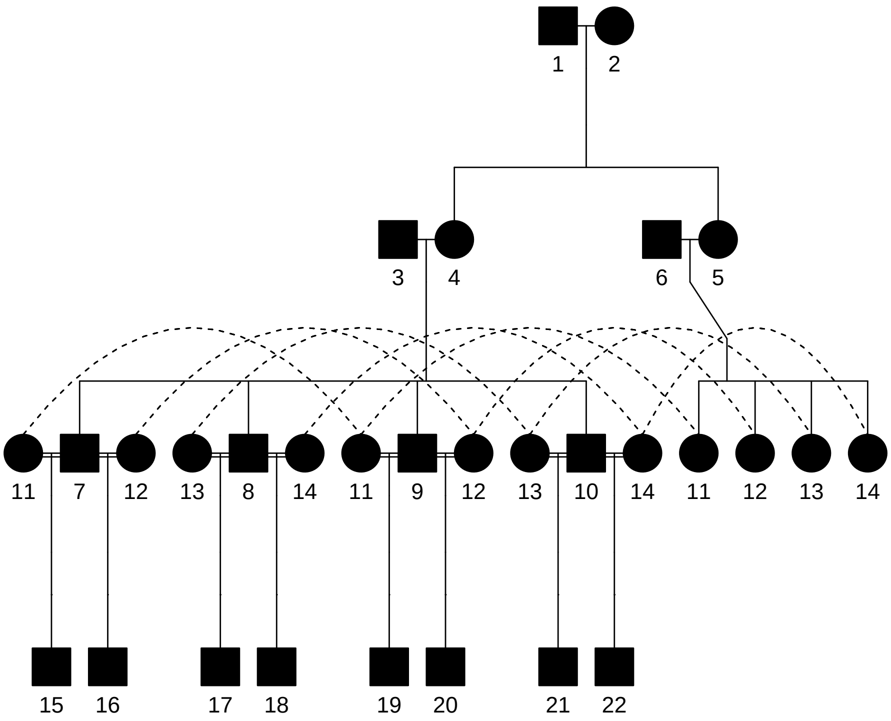

Supplementary Figure 2: An example with largest clique not less than 7. A total of 4 male and 4 female cousins each mate with 2 others in their generation. If there were 8 male and 8 female cousins each mating with 2 others, the complexity would grow linearly. However, if they were to mate with more than 2 others in their generation the complexity would grow exponentially.

Related Methods and Software

Here we compare RVS to other methods commonly employed for sequence data analyses in pedigrees: GESE, RareIBD, pVAAST and PBAT. Methods generally differ by aspects of experimental design, for example whether only affected family members are sequenced or both affected and unaffected are sequenced, and what type of information about variant frequencies is required. GESE, RareIBD and pVAAST were designed for analyses of rare variants in a gene (or genomic region in general), while RVS and PBAT were initially designed for single variants, but have options for region/gene based analyses. Scalability also differs substantially between the methods (see Table 3 in biorxiv 285874). Compared to the other methods, RVS offers some unique features. While allowing for phenocopies, RVS is specifically designed to only use genomic data from affected relatives. Further, no parental or founder data is required, which can be of great advantage, as commonly it is difficult to obtain DNA from past generations. RVS also prioritizes variants by a potential to achieve significance without affecting the type I error, greatly reducing

the multiple comparisons problem. RVS also offers functionality to address cryptic relatedness among founders. RVS does not depend on knowledge of variant frequencies (which can be of great advantage when a population is studied that is not well represented in public data bases), except that it has to be sufficiently rare that multiple founders introducing the variant in the pedigree is unrealistic (functionality to carry out sensitivity analyses is available).

RVS

https://bioconductor.org/packages/release/bioc/html/RVS.html

PMIDs 24740360, 24793288, and biorxiv 285874.

RVS is designed to detect very rare, highly penetrant variants. The frequency of these causal variants should be low in external reference data bases (such as gnomAD) and thus variants are typically pre-filtered based on frequency. The list of variants can be further restricted using variant annotation tools, for example only considering exonic variants predicted to be highly damaging or using only nonsense mutations. If a variant is indeed rare and identity-by-state (IBS) implies identity-by descent (IBD), then sharing by multiple affected relatives can represent a departure from the null hypothesis of no linkage or association. Thus, samples for sequencing are chosen only among affected family members, and large genetic distances between the relatives are desirable to have sufficient power in the inference. In particular, the unaffected parents of an affected subject are *not* used in the analysis (they might be typed for candidate variants to investigate the possibility of sequencing errors, e.g. PMID 24793288, Figure 1). Information from multiple families can be combined by calculating a p-value as the sum of the probabilities of sharing events as (or more) extreme. Only variants achieving a sufficiently low p-value for genome-wide significance if shared by all affected subjects in the family (or families) are tested. These "potential p-values" are independent of the actual sharing pattern among affected relatives, and therefore of the subsequent testing of variant sharing. RVS has recently been expanded to include gene-based analyses, a partial sharing test based on RV sharing probabilities for subsets of affected relatives (allowing for phenocopies) and a haplotype-based RV definition. RVS has also been used to examine sharing of rare copy number variants.

GESE

https://cran.r-project.org/web/packages/GESE/

PMID 28191685.

In contrast to RVS the gene-based segregation test (GESE) requires an estimate of variant frequencies to calculate an unconditional probability of segregation patterns (as compared to calculating the probability of sharing conditional on the variant being observed), but otherwise relies on very similar assumptions as RVS. Specifically, GESE (like RVS) assumes only one founder in the family introduced a causal variant in a gene, and the authors recommend limiting the tests to variants with high functional impact. Further, GESE also calculates the p-value as the sum of the probabilities of all events as or less likely as the observed event. However, in addition to absence of phenocopies GESE also assumes complete penetrance, while RVS is based only on

sharing among affected subjects, and does not make any assumptions about unaffected subjects. GESE uses the sequence data from affected and unaffected family members, but can be run in "affected-only" mode by setting the phenotype of unaffected subjects to unknown.

RareIBD

http://genetics.bwh.harvard.edu/rareibd/

PMID 27666371.

Another method that does not rely on control samples or external allele frequency estimates is the gene-based method RareIBD. Instead of using exact sharing probabilities, the test statistic is calculated as the sum of the number of affected subjects sharing a rare variant and the number of unaffected subjects without the rare variant (either of which can be zero, for example when only affected family members are sequenced), standardized by subtracting its expectation and dividing by its standard deviation within each family under the null hypothesis. Inference can then be based on an asymptotic standard Gaussian null distribution if a large number of family members are sequenced, or otherwise on a permutation null distribution. RareIBD was mainly devised for settings where founders are also sequenced (for multi-generation pedigrees this is often not the case, as commonly founder DNA is not available) but can also be employed without the sequence of founders, for instance when mostly distantly related family members are sequenced. As for RVS, the key assumption in RareIBD is that the causal variant is rare, in the sense that when it is seen identical by state (IBS) in multiple affected subjects it has to be identical by descent (IBD). Thus, variant filtering methods using reference databases have to be used to generate a list of rare variants to be analyzed. RareIBD and RVS coincide in the special case when single variants are analyzed, no founders are sequenced, and the pedigrees have only one affected relative pair of the same type such as an affected second cousin pair. In this case RVS calculates a p-value based on a closed-form, while RareIBD uses a Monte Carlo gene dropping procedure.

pVAAST

http://www.hufflab.org/software/pvaast/

PMID 24837662.

When reliable information about allele frequencies is available, combining a linkage signal with an association signal derived using ~~known~~ allele frequencies can increase statistical power. The pedigree Variant Annotation, Analysis and Search Tool is based on this notion, combining the pedigree linkage signal with a likelihood-ratio-based rare variant association test that incorporates case/control allele frequency differences and functional annotation into the likelihood. While greater power can indeed be achieved when all assumptions are met, the test is highly sensitive for example to variant frequency estimation. The pVAAST linkage LOD score alone generally has low power.

PBAT

https://www.hsph.harvard.edu/pbat/download2/

PMIDs 12214309, 15814068.

Family-based association tests (FBAT; Lange and Laird 2002, PMID 12214309) are a suite of tools extending the basic principle of the transmission disequilibrium test. PBAT is a more comprehensive software package that incorporates the features of the FBAT package, but can also handle extended pedigrees with missing genotypes, in addition to nuclear families (van Steen and Lange 2005, PMID 15814068). *"The cornerstone of PBAT is the unified approach to the FBAT statistic, which itself is a generalization of the Transmission Disequilibrium Test (TDT) method, in which alleles transmitted to affected offspring are compared with the expected distribution of alleles among offspring"* (doc.goldenhelix.com/SVS/latest/svsmanual/pbat.html). Mathematically, the FBAT statistic simply compares a linear combination of offspring genotypes and recorded traits to its expected value and variability. Contrary to RVS, FBAT/PBAT can be used for common and rare variants, and the alleles transmitted to affected offspring are compared with the expected distribution of alleles among offspring. Thus, sequencing should be considered for affected subjects and their parents to observe many such transmissions, (or unaffected siblings when parents are unavailable). FBAT/PBAT does not specifically target highly penetrant variants, or exploits the rarity of the variant in any way. There is a 'rare variant' version of FBAT, but this is a burden test based on collapsing variants in specific regions, with the option to weight the variants by their frequency (De et al 2013, PMID 23341868; Wang et al 2016, PMID 27980642).
